# When interlocutor’s face-language matching alters: An ERP study on face contexts and bilingual language control in mixed-language picture naming

**DOI:** 10.3389/fpsyg.2023.1134635

**Published:** 2023-03-23

**Authors:** Binyuan Zhuang, Lijuan Liang, Jing Yang

**Affiliations:** ^1^Bilingual Cognition and Development Lab, Center for Linguistics and Applied Linguistics, Guangdong University of Foreign Studies, Guangzhou, China; ^2^Faculty of English Language and Culture, Guangdong University of Foreign Studies, Guangzhou, China; ^3^School of International Studies, Zhejiang University, Hangzhou, China

**Keywords:** bilingual lexical production, face contexts, reactive language control, proactive language control, event-related potentials

## Abstract

The present study used event-related potentials (ERP) to examine Chinese-English bilinguals’ reactive and proactive language control as they performed mixed-language picture naming with face cues. All participants named pictures in Chinese (first language, L1) and English (second language, L2) across three sessions: a 25% face-language matched session, a baseline session without face cues, and a 75% face-language matched session. Behavioral analyses for reactive language control showed that the asymmetrical switch cost was larger for L2 than L1 in the 25% session and for L1 than L2 in the 75% session. ERP results revealed more negative N2 and LPC during L1 switching in 25% session but enhanced N2 during L2 switching in 75% session. Similar N2 and LPC effect was found during L1 and L2 switching in the baseline context. For proactive language control, the reversed language dominance and enhanced LPC amplitudes during L2 naming were consistent across the three sessions. Our findings suggest that reactive but not proactive language control is modulated by the ever-changing face contexts, which highlights the highly flexible bilingual control systems subserving nonlinguistic cues.

## 1. Introduction

Bilinguals are usually sensitive to interlocutors’ faces, which signal their language identity and prompt bilingual language selection (Woumans et al., 2015; Bhandari et al., 2020; Kaan et al., 2020). However, face cues could also be misleading and impede language production and communication (e.g., Zhang et al., 2013; Roychoudhuri et al., 2016). While an increasing body of studies investigates faces as a nonlinguistic cue in bilingual language processing, few examine how bilingual experiences shape the face priming effect (e.g., Woumans et al., 2015; de Bruin and Martin, 2022). Additionally, as bilingual language control involves reactive and proactive language control, it remains unclear how the two subsystems support bilingual lexical access in different face contexts.

### 1.1. Bilingual language control: Reactive and proactive language control

According to the Inhibitory Control (IC) model (Green, 1998), language control monitors and resolves the interference and competition between the two languages in bilinguals. The adaptive control hypothesis (Green and Abutalebi, 2013) highlights the influence of contexts on bilingual language control. It suggests that bilingual language control system adapts to different language contexts (e.g., single-language, dual-language, and dense code-switching contexts) with varying demands. Meanwhile, bilingual language control is not a unitary construct. It comprises two coexisting subsystems: reactive language control at the local level (hence *reactive language control*) and proactive language control at the global level (hence *proactive language control*; Christoffels et al., 2007; Green and Abutalebi, 2013; Ma et al., 2016; Seo and Prat, 2019; Li et al., 2021). The two types of language control may work together in accommodating different interactional contexts (Ma et al., 2016; Wu et al., 2018; Timmer et al., 2019). Reactive language control is assumed to inhibit the nontarget language only after the specific lexical items are activated. It deals with the interference from the unintended language when both languages have been activated. Proactive language control, on the other hand, could regulate the languages’ activation level even before activating these lexical items. It can be associated with the anticipation of speaking an intended language and preemptive avoidance of potential interference from the unintended language. Accumulating evidence revealed that both types of language control are at play during speech production. More importantly, they could potentially change and interact with each other depending on different interactional contexts (Peeters and Dijkstra, 2018; Timmer et al., 2019; Liu et al., 2022).

Mixed-language picture naming is a prevalent language-switching task measuring bilingual language control. In this task, bilingual participants are cued to name pictures in two languages (Meuter and Allport, 1999; Branzi et al., 2014; Wu et al., 2018; Liu et al., 2019). Reactive language control is measured by *switch cost*, which refers to the longer naming latencies or lower accuracy rates in switch trials (to name the picture or digit in a different language with the last picture) than in repeat trials (to name the picture or digit in the same language with the last one). By contrast, the proactive language control is indexed by the *reversed language dominance* effect^1^ in mixed-language contexts, which refers to slower naming responses or lower accuracy rates in the dominant language (usually first language, L1) than in the non-dominant language (usually second language, L2) in mixed-language contexts (Meuter and Allport, 1999; De Groot and Christoffels, 2006; Christoffels et al., 2007; Gollan and Ferreira, 2009; Bonfieni et al., 2019; Declerck et al., 2021; but see Ma et al., 2016).

According to the IC model (Green, 1998), during the language switching of the picture naming task, the dominant L1 requires greater inhibition to suppress those competing items during L2 access. Therefore, a switch cost asymmetry is often observed for unbalanced bilinguals, with a higher cost when switching to the dominant L1 from L2 than vice versa (Meuter and Allport, 1999; Verhoef et al., 2009). For balanced bilinguals, more symmetrical patterns are observed when bilinguals switch languages in both directions (Costa et al., 2006; Calabria et al., 2012). Nevertheless, unbalanced bilinguals have also reported symmetrical switch costs in several studies (Costa and Santesteban, 2004; Peeters and Dijkstra, 2018; Liu et al., 2019, 2021). The reversed language dominance effect suggests sustained proactive language control over the dominant L1 (Bobb and Wodniecka, 2013; Wu et al., 2018 for a review, see Declerck, 2020) during the mixed-language naming contexts. Bilinguals reply on the proactive control to counteract the strong activation of the dominant language preemptively and facilitate L2 access.

Studies using event-related potentials (ERPs) found distinct patterns for proactive and reactive language control in the bilingual brain. The reactive language control measured by switch cost has been associated with N2 (Jackson et al., 2001; Verhoef et al., 2009; Liu et al., 2020) and Late Positive Component (LPC; Martin et al., 2013; Liu et al., 2016, 2020). In language switching studies, the N2 component indicates that inhibition may take place during the language selection phase, while the LPC indicates that inhibition may occur during the lexical selection phase (Jackson et al., 2001; Liu et al., 2016; Jiao et al., 2022b). Moreover, ERP studies revealed that the LPC component is sensitive to the reversed language dominance effect, with enlarged amplitudes for L2 relative to L1 trials (Liu et al., 2016; Timmer et al., 2017, 2019).

Accumulating evidence suggests that reactive and proactive language control have distinct neural activation patterns in the supplementary motor area (SMA), anterior cingulate cortex (ACC), and left dorsolateral prefrontal cortex (DLPFC; Guo et al., 2011; Seo and Prat, 2019; Yuan et al., 2021). Increasing behavioral studies (Christoffels et al., 2007; Gollan and Ferreira, 2009; Wu et al., 2018) and neurophysiological research (Timmer et al., 2019; Wu et al., 2020; Zhang et al., 2021) suggested that the two types of language control work together for optimal performance in mixed-language contexts. For example, Christoffels et al. (2007) asked unbalanced German–Dutch bilinguals to name pictures in a single-language context of L1 (German), L2 (Dutch), or mixed-language context. Results showed significant switch costs and mixing costs (i.e., another indicator of proactive language control) in the mixed-language context, suggesting that both reactive and proactive inhibition are actively engaged in bilingual word production.

In order to achieve a new balance of equal lexical access for both languages, proactive and reactive language control may take charge alternatively. This enables bilinguals to function at their best when speaking in varied linguistic circumstances. For instance, Timmer et al. (2019) explored whether Dutch-English bilinguals adapt their language control system to two linguistic contexts differing in proportion of language employment. Specifically, the L1-dominant context (participants named 83% of filler pictures in L1) witnessed the symmetric switch cost and the reversed language dominance effect. It was accompanied by larger LPC for L2 trials, implying a sustained inhibition of L1 during L2 naming. By contrast, in the L2-dominant context (participants named 83% of filler pictures in L2), the switch cost turned out to be asymmetric, with slower responses when switching to L2. Overall, L1 and L2 naming remained constant in both RTs and LPC effects. Their results indicated that reactive and proactive language control adapts flexibly to the varying language frequency in the language context. Proactive language control may even overrule reactive language control to some extent. Sustained inhibition of the dominant L1 has been suggested to decrease the contextual needs for reactive inhibition in mixed-language settings (Peeters and Dijkstra, 2018). However, few studies have examined the dynamics of the two language control subsystems in bilingual mixed-language picture naming when presenting with face cues.

### 1.2. The adaptation of bilingual language control to face contexts

A growing body of literature has underlined the effect of visual cues on bilingual language processing, such as faces with ethical features (e.g., the Asian and Caucasian faces in Li et al., 2013), culture-laden icons (e.g., the Great Wall in China and Mount Rushmore in America in Zhang et al., 2013; national flags in Grainger et al., 2017), or objects with distinctive national characteristics (e.g., Korean soup and North American soup in Berkes et al., 2018; Chinese mailbox and Canadian mailbox in Jared et al., 2013). These visual cues embodied with language-associated information boost the availability of the associated language and are assumed to prompt bilinguals toward the congruent language during production.

Why do bilinguals tend to speak the interlocutor’s language? The “audience design” theory suggests that the speaker adjusts their speech to favor the listener for successful communication (Clark, 1996). Similarly, the “interactive alignment” hypothesis postulates that the speakers mimic each other in language patterns during the conversation (Pickering and Garrod, 2004). From the perspective of social psychology, bilinguals select the interlocutor’s language to increase in-group interaction and decrease the cross-group anxiety that might hinder communication (Fu et al., 2007).

Faces as a prominent visual cue influence language selection in bilingual language comprehension (Molnar et al., 2015; Martin et al., 2016) and production (Li et al., 2013; Blanco-Elorrieta and Pylkkänen, 2015; Woumans et al., 2015; Kapiley and Mishra, 2019; Peeters, 2020). For example, Li et al. (2013) asked Chinese–English bilinguals and English monolinguals to name pictures within colored frames held by an Asian or a Caucasian. The Chinese-English bilinguals named pictures in their first language (L1, Chinese) faster when presented with Asian faces (congruent condition) than with Caucasian faces (incongruent condition) and the baseline task without face cues. That congruence effect was associated with increased brain activity in the left middle frontal gyrus (MFG), inferior frontal gyrus (IFG), and anterior cingulate cortex (ACC). Interestingly, the English monolinguals named pictures faster in the Caucasian face condition (congruent condition) than in the baseline condition without face cues, as per the findings of young children using facial cues in L1 language acquisition (Weikum et al., 2007).

Bilinguals can update the interlocutor’s language profile by reflecting on the interlocutor’s language use. They can remove the previously attached language tag when the link between the faces and languages becomes inconsistent. Woumans et al. (2015) investigated the influence of the interlocutor in the lexical production of Spanish-Catalan and Dutch-French bilinguals. The participants were firstly familiarized with some interlocutors *via* Skype and then asked to speak to them in their corresponding language, thus creating a language-face association. After the exposure, the participants were asked to produce nouns associated with the verbs produced by interlocutors. Half of the interlocutors were familiar faces exposed to them in the initial phase, and the other half were unfamiliar faces. The familiar interlocutors produced words, half in the same language used in the initial exposure stage and half in a different language. The authors reported that their participants responded faster to congruent trials than incongruent trials at the beginning stage of the noun-verb associative task. However, this congruency or facilitation effect disappeared in later trials with increasing incongruent trials. Woumans et al. (2015) suggested that the reliability of face cues links with their influence on bilingual language selection. Nevertheless, the question remained elusive as to whether the mechanism of bilingual language control adapts to different nonlinguistic cues, especially face cues.

There has been little agreement on whether and how bilingual language control functions in nonlinguistic contexts, given limited evidence. Roychoudhuri et al. (2016) examined Bengali-English bilinguals’ reaction to Bengal cultural images (e.g., Howrah Bridge, cultural heritage of Bengal) and neutral images (e.g., banana) during picture naming. They assessed the switch cost and mixing cost (another indicator for proactive language control) in a mixed-language context of Bengali and English and two single-language Bengali/English contexts. The results revealed that cultural cues did not influence the reactive language control (indexed by switch cost) or proactive language control (indexed by mixing cost) compared to culturally neutral cues. The cultural icons were perhaps less salient than faces for language control, as bilinguals are less inclined to make language decisions when exposed to cultural symbols than face cues under the account of “interactive alignment” (Pickering and Garrod, 2004). Unlike faces, culturally embodied objects do not elicit language control mechanisms when one looks at them. Thus, their influence on the speaker’s language production seems more subtle than those of face cues (Som and Kalita, 2018).

There is a growing interest in the influence of face cues on bilingual language control. For instance, Liu et al. (2019), adopting the paradigm of Li et al. (2013), asked Chinese-English bilinguals to name pictures in the absence or presence of faces with socio-cultural identity (Asian or Caucasian faces). A switch cost asymmetry with a larger switch cost for L2 than L1 was observed in the congruent context (e.g., speaking Chinese after seeing Asian faces). The patterns differed from what was found in the incongruent context (e.g., speaking Chinese after seeing Caucasian faces) and the no-face baseline context. In both contexts, there were symmetrical switch costs (Experiment 1) and larger switch costs in L2 (Experiment 2). By contrast, the reversed language dominance effect remained constant across the three contexts. Therefore, the authors claimed that contextual faces modulate local but not global language control. Liu et al. (2021) replicated this study with similar reactive and proactive language control patterns using symbolic cultural icons as nonverbal cues. Critically, they included cultural icons of L1 and L2 (e.g., Chinese chess vs. western chess) in nonlinguistic contexts.

It is noteworthy that Liu et al. (2019) have tested bilingual language control mechanisms in 100% face-language matched and 100% unmatched contexts. In reality, this is not the case. For example, the face-language link in the L1 and L2 environments is not 100% congruent or incongruent. Whether and how language control adapts to face contexts with varying degrees of face-language mapping remains to be examined. More critically, the current study provides one of the first neurological investigations into the influence of facial contexts on the plasticity of bilingual language control.

### 1.3. The present study

The present ERP study investigated the influence of face contexts on bilingual reactive and proactive language control mechanisms in lexical production. The adaptive control hypothesis (Green and Abutalebi, 2013) postulates that processing contexts with different demands flexibly modulate the language control system. Our findings will shed new light on the relationship between facial context and language control (i.e., reactive and proactive language control). The dynamic interplay of proactive and reactive language control provides a broader venue for exploring the underlying language control mechanism. Unlike the 100% face-language matched or unmatched conditions in Liu et al. (2019), we manipulated the ratio of face-language congruency: a 25% face-language matched session and a 75% face-language matched session. A baseline session without face cues was included in the present study. All the participants completed the picture naming tasks in the three sessions. Based on previous behavioral and ERP findings, we expect reactive language control (switch cost) changes in the varying contexts but not proactive language control (reversed language dominance effect).

If reactive language control flexibly adapts to different face contexts, we expect distinct switch costs patterns, accompanied by different N2 and LPC effects during language switching. Face-language mapping would bias language activation such that language switching after viewing matched faces would be less costly than unmatched faces in both L1 (Chinese) and L2 (English). We would expect opposite patterns of asymmetrical switch cost between L1 and L2 in the two face contexts, with divergent N2 and LPC patterns during L1 and L2 switching. In the 75% face-language matched context, the face cues, which are relatively reliable non-linguistic cues, could make switching to the target language easier. However, as they became much less reliable in the 25% face-language matched context, these same faces might increase the burden of switching to the other language. In the baseline context with no face cues, based on previous studies in language switching (Jackson et al., 2001; Verhoef et al., 2009), we expect to find an asymmetrical switch cost larger for L1 than for L2 in unbalanced bilinguals, together with larger N2 and LPC for L2 switch trials compared to repeat trials. The proactive language control, indicated by the reversed language dominance effect, might be associated with a larger LPC in the L2 than the L1 (Timmer et al., 2019) in all three contexts.

## 2. Methods

### 2.1. Participants

Twenty-eight Chinese-English bilinguals (26 females, ages 19–25) participated in all three sessions of the current study. We used G^*^power 3.1.9.7 (Faul et al., 2009) to calculate the power of sample size. When the Effect size *f* was set at 0.25 (Liu et al., 2022) and α error probability set at 0.05, the power of our sample size was estimated to be above 0.95. Participants were healthy and right-handed undergraduates or graduates from the Guangdong University of Foreign Studies with normal or corrected-to-normal vision. All were English majors who began learning English as L2 around 8 years old (*SD* = 2.27). They were upper-intermediate to advanced bilinguals based on their performance in the Oxford Quick Placement Test, a standardized English proficiency test (Syndicate, 2001). They self-reported their proficiency levels for L1 and L2 in listening (L1: Mean = 6.21, *SD* = 0.83; L2: Mean = 4.38, *SD* = 0.82), speaking (L1: Mean = 5.88, *SD* = 0.90; L2: Mean = 4.04, *SD* = 1.04), reading (L1: Mean = 5.83, *SD* = 0.87; L2: Mean = 4.88, *SD* = 0.85), and writing skills (L1: Mean = 5.38, *SD* = 0.77; L2: Mean = 4.50, *SD* = 0.89) on a seven-point scale (1 for not fluent, 7 for very fluent) in the Language History Questionnaire (LHQ 3.0, Li et al., 2020). Among all these four skills, their ratings on L1 were significantly higher than L2 (all *t*s > 4.32, all *p*s < 0.001). It indicates that they were unbalanced bilinguals with L1 dominance. All participants provided informed consent and received monetary compensation upon completing the experiment. The present study was approved by the ethical committee of the Bilingual Cognitive and Development Lab at the Guangdong University of Foreign Studies, China.

### 2.2. Stimuli

The current experiment included a 25% face-language matched session, a 75% face-language matched session, and a baseline session without face cues. In all three sessions, participants were asked to name pictures in L1 (Chinese) and L2 (English). Those picture stimuli were colorless line drawings of familiar objects from the database of Snodgrass and Vanderwart (1980) and standardized by Chinese studies (Zhang and Yang, 2003; Liu et al., 2011). There were the same 160 picture stimuli in the three sessions of this experiment. The words for the 160 picture stimuli were provided in Supplementary Table S2. The pictures corresponded to high-frequency words (L1: Mean = 3.86, *SD* = 1.49; L2: Mean = 3.25, *SD* = 1.22, Bates et al., 2003). All word frequencies (Bates et al., 2003) in both languages were listed in Supplementary Table S3. The picture stimuli were presented in a pseudo-random order once in the baseline session and four times in the two sessions with face cues. Half of them should be named in L1 and half in L2. Of the trials within each language, half were switch trials (L2Switch trials: L1 → L2; L1Switch trials: L2 → L1), and the rest were repeat trials (L1Repeat trials: L1 → L1; L2Repeat trials: L2 → L2). There were 160 trials in the baseline session.

Four photos of Caucasian faces and four of Asian faces from the MR2 database (Strohminger et al., 2016) were used in the two sessions with face cues. Those photos comprised half males and half females for each race, well-controlled in lighting, positioning, hair, and makeup. The 25% face-language matched session consisted of 160 face-language matched trials (seeing Asian faces and speaking Chinese; seeing Caucasian faces and speaking English) and 480 face-language unmatched trials (seeing Asian faces and speaking English; seeing Caucasian faces and speaking Chinese). The 75% face-language matched session comprised 480 face-language matched trials and 160 unmatched trials. The face pictures of different races and gender were the same in 25 and 75% face-language matched sessions. The race and gender of the eight face pictures were counterbalanced within each session.

### 2.3. Task and procedure

Before the experiment, participants were familiarized with all the picture stimuli and the names of the pictures in L1 and L2 (Participants were given both the L1 and L2 names to get familiar with the pictures, but each picture was required to be named in only one language in the experiment). They completed a pre-test to name the pictures printed on a piece of paper and received feedback until there were no more mistakes. Participants completed the three sessions within a day or two consecutive days. In the baseline session, each trial began with a 500-ms fixation, followed by a red or blue frame as language cues for 300 ms and a 500-ms blank interval. Then a black-and-white drawing appeared in the center of the computer screen without the frame. The participants should overtly name the pictures according to the color of the frame (red for L1; blue for L2) as quickly and accurately as possible. The picture remained on the screen until the participant responded or after 1,000 ms. After a blank interval of 1,000 ms, the subsequent trial began. Naming latencies within 2,000 ms were collected.

**Figure 1:**
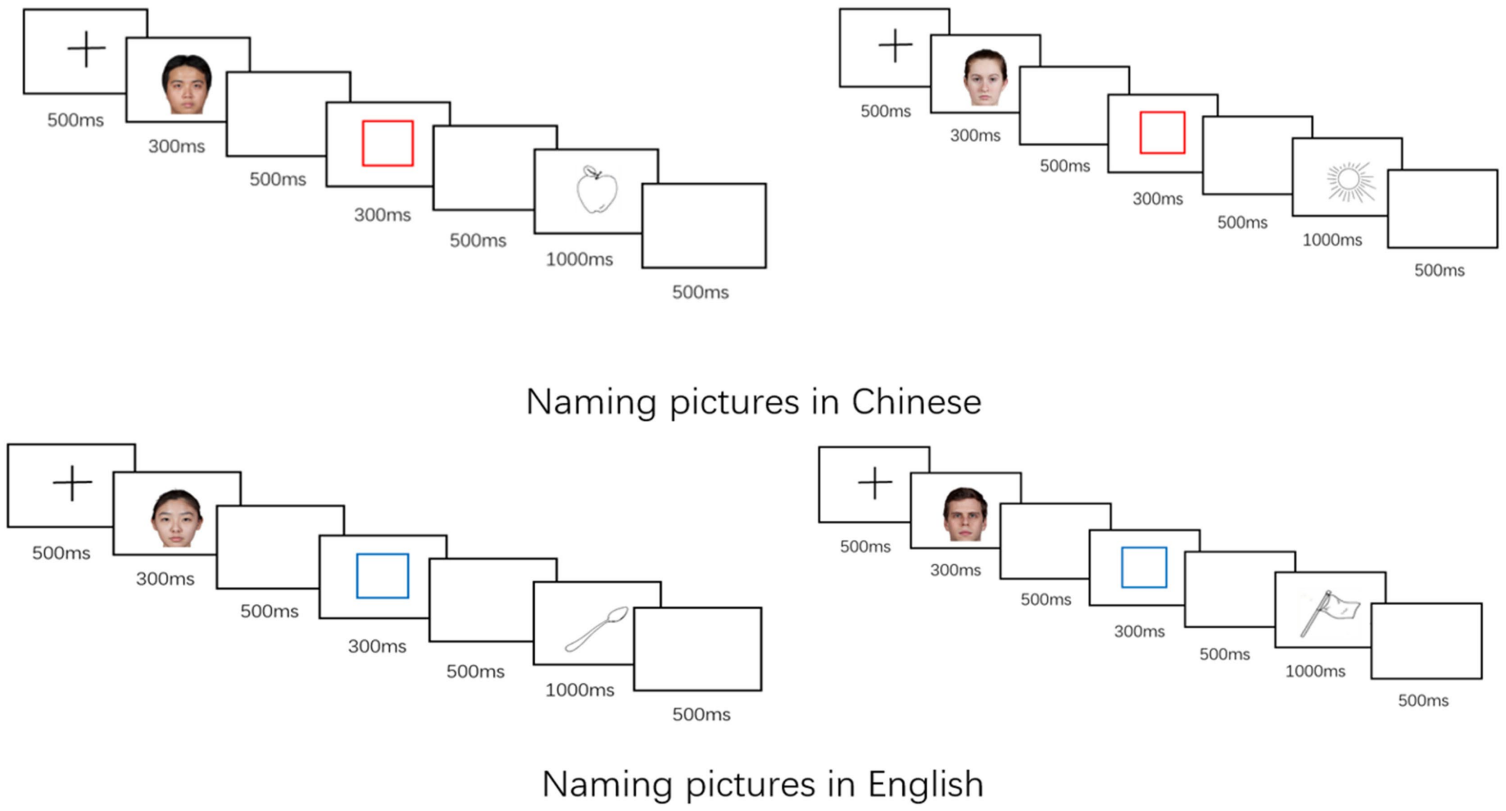
Task paradigm of mixed-language picture naming in the two face-language matched sessions (25% face-language matched session and 75% face-language matched session). There were two congruent conditions (speaking Chinese after viewing an Asian face; speaking English after viewing a Caucasian face) and two incongruent conditions (speaking Chinese after viewing a Caucasian face; speaking English after viewing an Asian face). In the 25% face-language matched session, the ratio of the congruent versus incongruent trials was 25%, while in the 75% face-language matched session, the ratio was 75%.

In the two sessions with face cues, each trial started with a 500-ms fixation, with a subsequent colored face photo of 300 ms, as well as a 500-ms blank interval. Then sequentially, the colored frame, the blank interval, and the target picture to be named showed up as in the baseline session (see Figure 1). Thirty-two catch trials (picture naming without a face cue) were randomly presented in the two sessions with face cues to keep the participants attending to the stimuli on the screen. To exclude possible interaction between the two sessions with face cues, we asked the participants to complete the baseline session after the 25% face-language matched session and before the 75% face-language matched session.

### 2.4. EEG recording and analyses

EEG signals were recorded by an electrode cap with 64 Ag/AgCl electrodes placed in line with the International 10/20 system, using NeuroScan Synamps2 (Compumedics, El Paso, TX, United States) with a sampling rate of 1,000 Hz. To monitor eye movements/blinks, we measured the veridical electrooculogram (VEOG) by two electrodes placed above and below the left eye, and the horizontal electrooculogram (HEOG) by two electrodes placed at the outer canthi of the eyes. All electrodes were referenced online to the right mastoid. The impedance of all electrodes was kept below 5 kΩ. EEG activity was filtered online with a bandpass between 0.05 and 100 Hz and digitally filtered offline at a bandpass of 0.1–30 Hz (12 dB setting). Artifacts induced by eye blinks were corrected through Independent Component Analysis (ICA) using EEGLAB (Delorme and Makeig, 2004; Brunner et al., 2013). The ERP grand averages were time-locked to the naming pictures and were computed independently for each participant in each condition. Continuous recordings were cut into epochs ranging from −200 to −800 ms after the onset of naming pictures. All ERP averages were aligned to a 200-ms baseline. Epochs with voltages exceeding ±100 μV in the EEG signals were discarded, along with incorrectly responded trials (altogether 14%). Data from four participants were excluded due to excessive EEG artifacts. All participants had at least 70% trials in each condition in all three sessions.

Considering the nature of the N2 and LPC components, together with the collected data in the current experiment, two temporal windows were chosen: 250–350 ms window for N2 and 450–650 ms window for LPC. Following previous ERP studies on language switching (Zhang et al., 2020; Jiao et al., 2022a), we analyzed the mean amplitude of the waveform across these two time windows of N2 (frontal: F1, FZ, F2; frontal-central: FC1, FCZ, FC2) and LPC (central: C1, CZ, C2; central-parietal: CP1, CPZ, CP2; parietal: P1, PZ, P2). We conducted a linear mixed-effects model for each time window for statistical analyses. This model included Session (baseline session without face cues, 25% face-language matched session, and 75% face-language matched session), Language (L1, L2), Trial type (Switch, Repeat), and their interactions as fixed effects, and participants as a random effect. We reported the results of *post hoc* analyses (with Tukey correction for multiple comparisons) using the emmeans package (Lenth et al., 2020) when the fixed effects reached a significant alpha level of 0.05.

The following ERP results were based on data from 24 participants in the baseline session, 26 in 25% face-language matched session, and 27 in 75% face-language matched session.

## 3. Results

### 3.1. Behavioral results

All the analysis was based on correct trials. Trials with voice-key errors or incorrect responses were removed from further analysis (See Supplementary Table S1 for details in Supplementary material). Naming latencies less than 150 ms, more than 1,500 ms, or above 2.5 SDs of the average response time per participant were also excluded. Another four participants were excluded for their limited available trials due to great voice-key errors or low accuracy rates in the picture naming task (less than 80% trials for data analysis). Therefore, in the current behavioral data analysis, there were 22 participants in the baseline session, 25 in the 25% face-language matched session, and 26 in the 75% face-language matched session. Table 1 summarizes participants’ response time and accuracy rates in the three sessions. We did not report the analysis of the accuracy rates data because of the ceiling effect (mostly above 95% for the three sessions) following previous studies (e.g., Ma et al., 2016; Wu et al., 2020).

**Table 1:** Averaged response time (ms) and accuracy rates (%; standard errors in parentheses) in three sessions.

Session	L1Switch	L1Repeat	L2Switch	L2Repeat	Switch cost		Reversed L1
					L1	L2	
Baseline (RT)	909 (23.3)	889 (22.6)	847 (19.4)	841 (19.2)	20	6	55
25% (RT)	987 (21.3)	975 (20.8)	938 (18.0)	915 (18.1)	12	23	54
75% (RT)	941 (23.3)	894 (22.6)	865 (19.0)	841 (18.7)	47	24	64
Baseline (ACC)	94.89 (1.79)	96.71 (0.66)	95.68 (0.74)	98.30 (0.52)	1.82	2.62	1.19
25% (ACC)	93.55 (1.15)	93.27 (0.82)	95.35 (0.74)	94.53 (0.62)	−0.28	−0.82	1.53
75% (ACC)	95.11 (0.81)	95.14 (1.02)	95.99 (0.49)	95.96 (0.70)	0.03	–0.03	0.85

Switch cost, measuring reactive language control, was calculated by subtracting response times (ms) of switch trials to repeat trials, and accuracy rates (%) of repeat to switch trials; reversed L1 (reversed language dominance effect), the indicator of proactive language control, was calculated by subtracting mean response times of L1 trials to L2 trials and accuracy rates of L2 to L1 trials. Baseline, baseline session with no faces; 25%, 25% face-language matched session; 75%, 75% face-language matched session. RT, response time; and ACC, accuracy rate.

We conducted a linear mixed-effects model on RTs, with Session (baseline session, 25% face-language matched session, and 75% face-language matched session), Language (L1, L2), Trial type (Switch, Repeat), and their interactions as fixed effects, and participants and items as random effects. The Session factor was helmert coded, so that the first contrast (Session_ratio_) displayed the difference between the 25% face-language matched session and the 75% face-language matched session, and the second contrast (Session_face_) reflected the difference between the face-language matched sessions and the baseline session. This approach allows us to examine the differences between the two sessions with varied face-language mappings and assess the difference between the sessions with and without face-language mapping. The other factors were coded using contrast coding (i.e., L1 = −0.5, L2 = 0.5; Switch = −0.5, Repeat = 0.5), yielding analogous tests of the main effects to ANOVA. To improve the RT model parameters, we included by-participant and by-item random intercepts, by-participant random slopes for Session, Language, and Trial type, and by-item random slopes for Language. The random slopes for the other factors and interactions were excluded due to convergence issues (Barr et al., 2013).

Table 2 shows a significant main fixed effect of Session_ratio_, indicating a slower naming latency in the 25% face-language matched session than in the 75% session. The main effect of Session_face_ was also significant, suggesting a faster naming latency for the no-face baseline session than for the other two sessions with face cues. For reactive language control indicated by switch cost, there was a significant main effect of Trial type, suggesting a switch cost with overall slower RTs in Switch trials than in Repeat trials. The interaction between Language and Trial type did not reach significance. The interaction between Session_ratio_ and Trial type, as well as the three-way interaction of Session_ratio_, Language, and Trial type were significant, suggesting discrepant patterns of switch cost between L1 and L2 across the two sessions with faces.

**Table 2:** Mixed-effects model for RT.

Fixed effects	Estimate	*SE*	*t*	*p*
(Intercept)	903.54	18.05	50.06	**<0.001** ^***^
Session_ratio_	−34.28	4.12	−8.31	**<0.001** ^***^
Session_face_	−16.09	3.66	−4.40	**<0.001** ^***^
Language	−57.57	12.21	−4.72	**<0.001** ^***^
Trial type	−22.02	4.81	−4.58	**<0.001** ^***^
Session_ratio_: Language	−4.99	2.93	−1.70	0.089
Session_face_: Language	1.34	2.36	0.57	0.569
Session_ratio_: Trial type	−9.01	3.58	−2.51	**0.012** ^*^
Session_face_: Trial type	4.28	2.46	1.74	0.083
Language: Trial type	8.35	7.68	1.09	0.277
Session_ratio_: Language: Trial type	17.03	7.11	2.40	**0.017** ^*^
Session_face_: Language: Trial type	2.74	4.9	0.56	0.577

Model formula for RTs: RT ~ Session × Language × Trial type + (1+ Session + Language + Trial type|Subject) + (1 + Language|Item).

Session_ratio_ compared the two sessions with faces (25% face-language matched session and 75% face-language matched session), and Session_face_ compared the two sessions with faces and the baseline session with no faces.

Significant effects were marked in bold. ^*^p < 0.05, ^**^p < 0.01, and ^***^p < 0.001.

Further explorations showed that in the 25% face-language matched session, Switch trials elicited longer RTs than Repeat trials only in L2 (*t* = 2.92, *p* = 0.019) but not in L1 (*t* = 1.565, *p* = 0.40), yielding an asymmetrical switch cost (L2 > L1; see Table 1). In the 75% face-language matched session, RTs in switch trials were longer than repeat trials in both L1 (*t* = 6.48, *p* < 0.001) and L2 (*t* = 3.09, *p* = 0.012), and the asymmetrical switch cost pattern in the 25% session was reversed in this session (L1 > L2). The interaction of Session_face_, Language, and Trial type did not reach significance, and in baseline session with no faces, Switch and Repeat trials did not differ significantly in L1 (*t* = 1.85, *p* = 0.252) and L2 (*t* = 0.59, *p* = 0.935). For *proactive language control*, the main fixed effect of Language reached significance, suggesting a reversed language dominance effect with slower naming in L1 compared to L2. The nonsignificant interactions between Language and Session_ratio_ as well as Language and Session_face_ indicated similar reversed language dominance effects between the three sessions (*ps > 0*.05).

### 3.2. ERP results

#### 3.2.1. N2 (250–350 ms time window)

**Figure 2:**
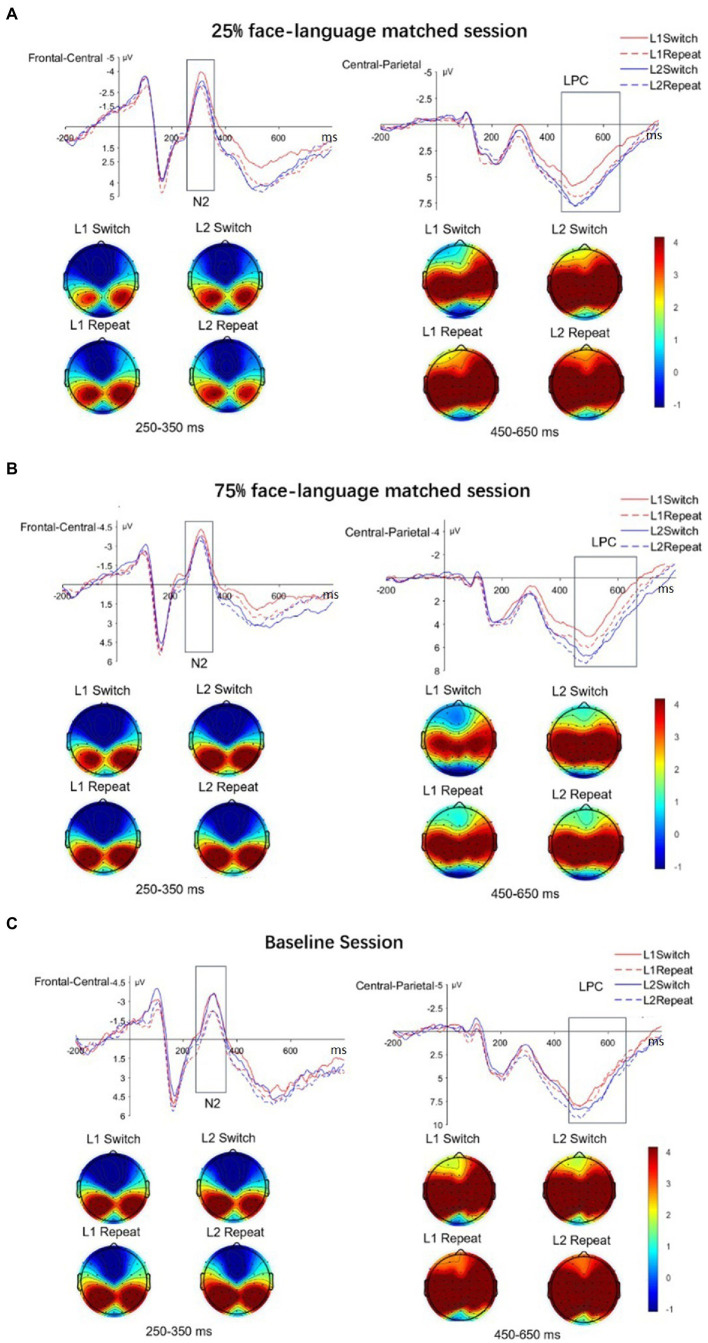
The grand average waveforms and topographic maps for **(A)** 25% face-language matched session, **(B)** 75% face-language matched session, and **(C)** baseline session per Language and Trial type. The upper panel displays grand average waveforms time-locked to the onset of the stimulus pictures for the two Trial types (Switch, Repeat) across two languages (L1, L2) in frontal-central and central-parietal regions. Red = L1; blue = L2; solid line = Switch; dotted line = Repeat. The lower panel exhibits topographic maps for each condition in the frontal-central and central-parietal regions.

Figure 2 displayed the grand average ERP waveforms during participants’ picture naming in the three sessions, respectively. As shown in Table 3, for reactive language control, the fixed effect of Trial type reached significance, with a larger N2 effect for Switch than Repeat trials. There was a significant interaction between Language and Trial type. Crucially, the interaction between Session_ratio_, Language, and Trial type also reached significance, suggesting different neural patterns in the two face-language matched sessions during L1 and L2 switching. Further analysis showed that in the 25% face-language matched session, only L1 Switch trials elicited larger N2 than Repeat trials (*t* = 4.17, *p* < 0.001) but not L2 (*t* = −0.08.17, *p* = 0.10; see Figure 2A). By contrast, in the 75% face-language session, larger N2 in Switch trials than Repeat trials was found only in L2 (*t* = −2.71, *p* < 0.001) but not in L1 (*t* = −1.70, *p* = 0.337; see Figure 2B). The interaction of Session_face_, Language, and Trial type did not reach significance, and in the baseline session, Switch trials elicited more negative N2 compared to Repeat trials in both L1 (*t* = −3.04, *p* = 0.023) and L2 (*t* = −2.50, *p* = 0.080; see Figure 2C). Therefore, the neural switch cost patterns varied among different sessions: it was asymmetrical (25% face-language matched session: L1 > L2; 75% face-language matched session: L1 < L2) in the two sessions with faces and symmetrical in the no-face baseline session. There was a significant effect of Language, indicating a larger N2 for L1 compared to L2 trials. The interaction between Language and Session_ratio_ reached significance. Further analysis showed that L1 trials elicited larger N2 compared to L2 trials in the 75% face-language matched session (*t* = −6.22, *p* < 0.001). The difference between L1 and L2 was not significant in the 25% face-language matched session (*t* = −0.85, *p* = 0.40) and baseline session (*t* = −0.66, *p* = 0.511).

**Table 3:** Mixed effects model for N2 and LPC.

	N2				LPC			
Fixed effects	Estimate	*SE*	*t*	*p*	Estimate	*SE*	*t*	*p*
(Intercept)	−1.68	0.63	−2.65	**0.013** ^*^	5.68	0.61	9.37	**<0.001** ^***^
Session_ratio_	−0.09	0.26	−0.36	0.723	−0.30	0.40	−0.75	0.459
Session_face_	0.19	0.16	1.17	0.253	0.43	0.27	1.59	0.126
Language	0.26	0.07	3.90	**<0.001** ^***^	1.25	0.24	5.22	**<0.001** ^***^
Trial type	0.74	0.19	3.93	**<0.00** ^***^	0.48	0.08	6.01	**<0.001** ^***^
Session_ratio_: Language	0.26	0.07	3.75	**<0.001** ^***^	0.20	0.16	1.27	0.216
Session_face_: Language	−0.08	0.05	−1.54	0.124	−0.15	0.11	−1.40	0.174
Session_ratio_: Trial type	0.05	0.13	0.38	0.710	−0.07	0.08	−0.81	0.416
Session_face_: Trial type	0.22	0.14	1.59	0.125	−0.09	0.06	−1.35	0.178
Language: Trial type	−0.29	0.13	−2.18	**0.030** ^*^	−0.82	0.16	−5.15	**<0.001** ^***^
Session_ratio_: Language: Trial type	0.58	0.14	4.17	**<0.001** ^***^	0.00	0.17	−0.01	0.994
Session_face_: Language: Trial type	0.03	0.11	0.28	0.778	0.48	0.13	3.74	**<0.001** ^***^

Model formula for N2 amplitude: Amplitude ~ Session × Language × Trial type + (1 + Session × Trial type|Subject).

Model formula for LPC amplitude: Amplitude ~ Session × Language × Trial type + (1 + Session × Language|Subject).

Session_ratio_ compared the two sessions with faces (25% face-language matched session and 75% face-language matched session), and Session_face_ compared the two sessions with faces and the baseline session with no faces.

Significant effects were marked bold. ^*^p < 0.05, ^**^p < 0.01, ^***^p < 0.001.

#### 3.2.2. LPC (450–650 ms time window)

As shown in Table 3, for reactive language control, there was a main effect of Trial type, with a larger LPC effect for Repeat trials than for Switch trials. The interaction between Language and Trial type reached significance, and there was a significant three-way interaction of Session_face_, Language, and Trial type, suggesting different ERP patterns when switching languages between the sessions with and without faces. The interaction of Session_ratio_, Language, and Trial type was not significant. Although this three-way interaction did not reach significance, further analyses were conducted in order to investigate switch cost patterns between the two sessions with faces. The results showed that in both the two face contexts, Repeat trials elicited more positive LPC than Switch trials in L1 (*t* = −7.70, *p* < 0.001 in 25% face-language matched session; *t* = −7.03, *p* < 0.001 in 75% face-language matched session). We did not find similar effects between L2 Switch and Repeat trials (*ps > 0*.05) in the two sessions with face cues. The baseline session showed no significant LPC difference between Switch and Repeat trials in both languages (*ps > 0*.05).

*Proactive language control* was reflected in the significant main effect of Language, which suggested a reversed language dominance effect. There was no significant interaction between Language and Session_ratio_ as well as Language and Sesssion_face_, suggesting that the reversed language dominance effect was stable across the three sessions.

## 4. General discussion

The current study investigated how face contexts influenced reactive and proactive language control during bilingual lexical production. Reactive language control, measured by the switch cost in the mixed-language picture naming task, is associated with N2 and LPC components of ERP data. Proactive language control, indicated by the reserved language dominance effect, is also connected with the LPC component. Larger N2 and LPC amplitudes for switch trials than for repeat trials are suggested to reflect switch cost (Jackson et al., 2001; Verhoef et al., 2009), while increased LPC in L2 than in L1 is associated with the reversed language dominance effect (Liu et al., 2016; Timmer et al., 2019; Jiao et al., 2022b).

For reactive language control, we found a larger switch cost in L2 in the 25% face-language match context, accompanied by more negative N2 and LPC in L1 switch trials relative to repeat trials. By contrast, there was a larger switch cost in L1 in the 75% face-language matched context, together with larger N2 in L2 switch trials relative to repeat trials. In the baseline session with no faces, we found no significant switch cost at the behavioral level but larger N2 on the switch than repeat trials in both L1 and L2 in the ERP data. Noticeably, instead of larger LPC, we constantly observed larger negativity for switch trials than repeat trials in the later time window. This pattern of the late component has been reported as switch-related negativity in several recent studies and is explained as increased difficulty in retrieving word meaning during language switching (e.g., Kang et al., 2020; Peeters, 2020; Declerck et al., 2021). For proactive language control, we consistently found a reversed language dominance effect as well as a larger LPC in L2 naming than L1 in all three sessions. The results suggested that consistency of face cues could influence reactive language control, while proactive language control remained relatively stable in contexts with or without face cues. A summary of the two language control patterns across the three contexts was presented in Supplementary Table S4 of the supplementary material. The following discussion elaborates on our findings and possible implications in detail.

### 4.1. Bilingual reactive and proactive language control: With and without face cues

The current study showed that the reactive language control altered in different contexts with and without face cues. It is indicated by the discrepant patterns of switch cost, as well as N2 and LPC components elicited by language switching in the two face sessions compared with the baseline session.

The IC model (Green, 1998) proposed that bilinguals applied inhibition to the non-target language in proportion to the activation level to resolve language competition. For unbalanced bilinguals, the dominant language receives more inhibition as it was more accessible relative to the non-dominant language. The extra inhibition could lead to asymmetrical switch cost (L1 > L2) at the local level and reversed language dominance at the global level, together with larger N2 and LPC when switching to L2 and larger LPC in overall L2 naming. The pattern of reversed language dominance with slower L1 naming in the baseline context was consistent with previous language-switching studies for unbalanced bilinguals (e.g., Christoffels et al., 2007; Jiao et al., 2022b). At the neurological level, larger LPC for L2 trials, on the whole, is in line with preceding ERP studies of language switching (Liu et al., 2016; Timmer et al., 2019; Peeters, 2020), which highlighted global inhibition of the dominant L1 to facilitate efficient production in both languages in a mixed-language context. The sustained inhibition of the dominant L1 also resonates with a recent dual-brain EEG study of simultaneous production and comprehension (Liu et al., 2022), which showed increased delta synchronization in mixed-language contexts and single-L2 context compared to a single-L1 context.

Although switching costs in the baseline session did not reach statistical significance in behavioral performance, the N2 effect revealed significant switch costs between switch and repeat trials. The inconsistent behavioral and ERP switch cost patterns have also been found in Jiao et al. (2022a). As ERPs are time-sensitive, they permit the separation of different processing phases through certain components and thus provide more detailed information, in contrast to an aggregation of various cognitive processes represented in RTs (Kang et al., 2020; Jiao et al., 2022a). The ERP patterns revealed symmetrical neural switch cost with a larger N2 effect in switch trials relative to repeat trials in both L1 and L2, which parallels with what was found for unbalanced bilinguals in a recent study (Peeters and Dijkstra, 2018). Likewise, several other studies have challenged the asymmetrical switch cost patterns for unbalanced bilinguals. For example, Liu et al. (2019) and Liu et al. (2021) reported symmetric switch costs in similar baseline contexts with no cultural cues.

However, in the two face sessions, we found distinct patterns of asymmetrical switch costs between L1 and L2. A larger switch cost in L1 than in L2 was found in the 75% face-language matched context, whereas a larger L2 switch cost than L1 was found in the 25% face-language matched context. These results indicate that the face contexts may come into play during language switching and modulate local reactive language control. As is documented in previous literature on bilingual language processing, the activation levels of the two languages are likely to change as a function of the face cues in a mixed-language context. Specifically, face cues facilitate language production when the face-language association coincides with the speaker’s expectations (Li et al., 2013; Woumans et al., 2015; Liu et al., 2019; for a review, see Hartsuiker, 2015). The results of the current study revealed that the influence of face contexts could be extended to the plasticity of bilingual language control.

As for proactive language control, consistent with our predictions, we observed the reversed language dominance effect of RTs (Liu et al., 2019, 2021), with an enlarged LPC for L2 trials than for L1 trials in all three contexts. It is in line with the preceding ERP studies of language switching (Liu et al., 2016; Timmer et al., 2019; Peeters, 2020). The consistent patterns across the three contexts suggested that the global proactive language control was less resilient to the perception of face cues.

Overall, our results showed the collaborative functioning of the two types of bilingual language control in mixed-language contexts. It suggested that bilinguals’ adaptation in varying face contexts manifests in reactive language control, which is consistent with previous research (Liu et al., 2019).

### 4.2. Bilingual reactive and proactive language control: The reliability of face cues

Given that the perception of face cues is at play in modulating reactive language control at the local level, the potential effect of face-language consistency is still open to question. The contrast of the results between two sessions with faces allows us to dive into the impact of face cues more intensively: whether the language control system is susceptible to the reliability of face contexts with varying degrees of face-language mapping (i.e., 25 vs. 75%). Not surprisingly, we found that reactive language control responded to the increase in the reliability of face cues. It is suggested by the reversed asymmetry of switch cost between L1 and L2 and distinct N2 and LPC patterns during L1 and L2 switching in the two face contexts.

According to the IC model (Green, 1998), bilinguals need to suppress the non-target language in order to minimize cross-language interference and successfully access words in the target language during language switching. We speculate that face cues may promote the selection of the lexical representations of the intended language when there is consistent face-language mapping. The IC model (Green, 1998) proposed that the dominant language needs to be inhibited to a larger extent than non-dominant L2. Thus, a suspension is induced to reactivate the dominant language when switching back to it after speaking in L2. Nevertheless, this is not the case in a context with strikingly low face-language mapping. In the present study, it seems that Caucasian faces capture participants’ attention more than Asian faces. Specifically, Caucasian faces are prone to boost the activation of L2, making it more effortful to switch back to the dominant L1 after viewing the faces than switching back to L2 with the presence of Asian faces. Compared to switching to L2 (English) after viewing Asian faces, switching to L1 (Chinese) after the perception of the Caucasian faces requires more inhibition of L2. It is due to the spreading activation of the non-dominant language from the Caucasian faces, combined with the residual activation of L2 from the previous trial. This enhanced inhibition of L2 was reflected in increased negativity in N2 and LPC components in L1 switch trials relative to repeat trials in the 25% face-language matched context. This unique pattern of asymmetrical switch cost was also observed in a linguistic context where L2 was used at an overwhelmingly high rate of 83%, and the lexical representations of the non-dominant L2 became more accessible (Timmer et al., 2019). It was the same rationale when the naming cue and stimulus interval was long enough to activate both languages to a similar degree (Ma et al., 2016).

In contrast to what was found in the 25% face-language matched context, L1 switch cost in the 75% face-language matched context was observed to be larger than L2, accompanied by more negative amplitude in N2 during L2 switching. As the faces were highly consistent with the naming cue in this context, when participants switched to L2 after viewing the Caucasian face, the lexical representations in L2 were reactivated in advance. It became less costly to reactivate the previously suppressed L2 to achieve successful speech production, yielding a smaller switch cost in L2 than in L1.

Face cues with socio-cultural information have been revealed to facilitate speech production when they matched the language to be spoken (Li et al., 2013; Hartsuiker, 2015; Woumans et al., 2015). However, a consensus has not been reached regarding whether there is a bias toward different cultural-symbolic cues. Berks et al. (2018) have found a similar facilitatory effect from Korean and North American cultural icons to the corresponding language of L1 (Korean) and L2 (English) for Korean-English bilinguals. Liu et al. (2019), however, assumed that there was an own-race advantage in cue-language integration. In a congruent context where the face cues completely corresponded to the naming language (e.g., naming in Chinese after the presence of an Asian face), there was an asymmetrical switch cost (i.e., reduced switch cost in L1) during picture naming. They ascribed it to a stronger priming effect from the Asian faces to the dominant language, making it easier to re-activate the strongly suppressed L1 when switching back to it.

Unlike Liu et al. (2019), other-race Caucasian faces seemed to enjoy more advantages during language-cue integration in the current study. The discrepant findings between Liu et al. (2019) and the present study may arise from the two sides of the same coin. The own-race bias shows people are generally better at recognizing faces of their own race compared to faces of different races, while the other-race categorization advantage leads to faster responses to other-race faces than to own-race faces. This advantage has been found in Caucasians (Levin, 1996; Caharel et al., 2011) and Chinese participants (Feng et al., 2011; Zhao and Bentin, 2011). Despite that Chinese-English bilinguals may be more sensitive to the details of Asian faces, Caucasian faces are more likely to catch their interest, leading to a greater boost in the activation of L2. Unlike the block designs in Liu et al. (2019), the mixed design of congruent and incongruent trials in the face sessions might prompt our Chinese-English participants to be more sensitive to Caucasian faces as a strategy for picture naming in their L2, the less dominant language.

There was a possibility that participants may have built new connections between the faces and the languages in a context when they become aware of the unreliability of the face cues in the 25% consistent context. In that case, the Caucasian faces were associated with L1 (Chinese) while Asian faces were bound up with L2 (English). The bias for Asian faces may lead to a reduced switch cost of L2, as these face cues could facilitate the reactivation of greatly inhibited L2 when switching back to it. However, the larger switch cost of L2 of runs against this assumption. Conversely, more attention to Caucasian faces could result in stronger L1 activation, thus releasing the burden when switching back to L1 after viewing Caucasian faces. This behavioral pattern was in line with the smaller switch cost in L1. Nevertheless, it remained unclear why stronger inhibition was exerted on L2 with enhanced negative amplitudes when switching to L1. Therefore, we find the explanation in terms of conventional mapping between faces and languages (i.e., Asian faces with L1 and Caucasian faces with L2) more compelling.

For proactive language control, the reversed language dominance effect and larger LPC in L2 naming were observed in the two face contexts, which indicated that face contexts with changing face-language consistency did not restrain the global inhibition of the dominant language. The result of the current study is in line with previous evidence that global language control tended to be unaffected by nonlinguistic cues (Roychoudhuri et al., 2016; Liu et al., 2019, 2021).

In sum, when exposed to a real-life simulated context where the language profile of the interlocutor is not entirely predictable, the transient reactive language control is modulated by the changing associations between face cues and language. In contrast, sustained proactive language control is kept relatively intact.

### 4.3. Limitations and implications for bilingual language control system

We acknowledge that the current study contains some limitations. Firstly, the sequence of the sessions (contexts) was not counterbalanced across participants. To control for the influence of face context on each other, we separated the two face sessions with a baseline session without face cues. Second, a different number of participants were excluded in the three sessions due to excessive EEG artifacts and invalid voice-key responses. As we focus on the factors of face context, language, and trial type (switch, repeat) in the present study, we also fail to examine the congruency effects in detail. For example, the switching between congruent and incongruent trials in each session needs to be examined in future studies with more participants and trials. Given enough trials, it is also meaningful to examine the face-language congruency effect at the different phases within each context.

Consistent with the adaptive control hypothesis (Green and Abutalebi, 2013), the current study extended the findings of context influence on bilingual language processing (Olson, 2016; Timmer et al., 2019) and nonlinguistic processing (e.g., Yang et al., 2018). Going beyond the plasticity of language control to various linguistic contexts (Peeters and Dijkstra, 2018; Liu et al., 2022), it highlights faces as an important nonlinguistic cue in bilingual language processing and reveals the underlying language control mechanisms that adapt flexibly to contexts. Specifically, the proactive language control in bilinguals function stably in different contexts; the reactive language control is modulated when the face cue’s reliability in different context varies. Compared to the alternating roles of reactive and proactive language control in different linguistic contexts (Peeters and Dijkstra, 2018; Timmer et al., 2019), this research provides a possibility that the two types of language control may coexist and collaborate together to accommodate different face contexts.

Despite discrepant self-reported L1 and L2 performances, compared to Liu et al. (2019) and Liu et al. (2021), participants in the current study were upper-intermediate to advanced bilinguals based on Oxford Quick Placement Test scores. However, the existing language control studies examining nonlinguistic contexts were limited to bilinguals mainly exposed to the L1 environment. Whether and how reactive and proactive language control react to face context for bilinguals immersed in L2 (e.g., English) environment calls for further examination. Moreover, how the brain networks for reactive and proactive language control interact and adapt in the changing face contexts needs further neuroimaging explorations.

## 5. Conclusion

To conclude, the current ERP study investigated the proactive and reactive language control mechanisms during bilingual lexical processing in contexts with different reliability of face cues. Our findings demonstrate that unbalanced Chinese-English bilinguals could identify and take advantage of the nonlinguistic face cues. Reactive language control system adapts to contexts with the presence of face cues. It also has the potential to detect and accommodate the reliability of associations between face cues and language membership. By contrast, proactive language control remains constant in changing face contexts. Our study highlights that the bilingual language control system is highly flexible and adaptive to contexts embodied with nonlinguistic cues.

## Data availability statement

The raw data supporting the conclusions of this article will be made available by the authors, without undue reservation.

## Ethics statement

The studies involving human participants were reviewed and approved by ethical committee of the Bilingual Cognitive and Development Lab at the Guangdong University of Foreign Studies, China. The participants provided their written informed consent to participate in this study.

## Author contributions

JY, BZ, and LL designed the study and wrote and revised the manuscript. BZ conducted the experiments and collected data. ZB, JY, and LL wrote and revised the manuscript. All authors contributed to the article and approved the submitted version.

## Funding

This work was supported by a grant from the National Natural Science Foundation of China [3210070856] awarded to Dr. Lijuan Liang, and research grants (BCD202101, BCD202105) from the Bilingual Cognition and Development Lab, Center for Linguistics and Applied Linguistics, Guangdong University of Foreign Studies, and the Fundamental Research Funds for the Central Universities, China.

## Conflict of interest

The authors declare that the research was conducted in the absence of any commercial or financial relationships that could be construed as a potential conflict of interest.

## Publisher’s note

All claims expressed in this article are solely those of the authors and do not necessarily represent those of their affiliated organizations, or those of the publisher, the editors and the reviewers. Any product that may be evaluated in this article, or claim that may be made by its manufacturer, is not guaranteed or endorsed by the publisher.

## Footnotes

^1^Proactive language control could also be indexed by mixing cost and blocked language dominance effect (for a review, see Declerck, 2020). The current study adopted reversed language dominance effect to indicate proactive language control instead of mixing cost, as pure language contexts of L1 and L2 was not included due to overlong period of time (participants took approximately an hour to finish the whole experiment). The reversed language dominance effect was considered a reliable marker in mixed-language blocks (e.g., Christoffels et al., 2007; Liu et al., 2019), and served as an effective marker for the participants in the current study.
