# Supplementary material for: When interlocutor’s face-language matching alters: An ERP study on face contexts and bilingual language control in mixed-language picture naming
Source: Front Psychol. 2023 Mar 23;14:1134635. doi: 10.3389/fpsyg.2023.1134635 (PMC10078986; doi:10.3389/fpsyg.2023.1134635)
Supplement: Supplementary file 1 [file Data_Sheet_1.docx]

**Appendix A**

**Table S1**

*The deletion rates in the three sessions (baseline session with no face, 25% face-language matched session and 75% face-language matched session)*

| Criteria | baseline session | 25% matched session | 75% matched session |
| --- | --- | --- | --- |
| voice-key error | 4.43% | 5.34% | 3.14% |
| Incorrect responses | 3.61% | 5.96% | 4.45% |
| <150 ms; >1500 ms | 3.32% | 6.70% | 4.56% |
| beyond 2.5SD | 1.89% | 1.64% | 1.94% |

**Table S2**

*The name of pictures in the three sessions*

| **Chinese (L1)** | | |  |  | **English (L2)** | | |
| --- | --- | --- | --- | --- | --- | --- | --- |
| 杯子 | 镜子 | 刷子 |  |  | Arrow | ear | needle |
| 冰箱 | 酒瓶 | 松鼠 |  |  | balloon | elephant | nose |
| 草莓 | 拉链 | 锁 |  |  | banana | envelope | pants |
| 叉子 | 老虎 | 台灯 |  |  | basket | fence | pear |
| 插头 | 铃铛 | 太阳 |  |  | bed | fish | pencil |
| 茶杯 | 领带 | 兔子 |  |  | belt | flag | piano |
| 车轮 | 鹿 | 乌龟 |  |  | bicycle | foot | pineapple |
| 衬衫 | 蚂蚁 | 五角星 |  |  | boat | fox | Pumpkin |
| 尺子 | 猫 | 五线谱 |  |  | book | frog | radio |
| 大衣 | 帽子 | 西瓜 |  |  | bread | glasses | scissors |
| 袋鼠 | 门 | 虾 |  |  | broom | goat | seahorse |
| 蛋糕 | 拇指 | 小丑 |  |  | butterfly | guitar | seal |
| 地球 | 鸟 | 熊 |  |  | button | gun | shoe |
| 电话 | 女孩 | 雪人 |  |  | candle | hammer | snail |
| 房子 | 盆 | 鸭子 |  |  | car | helicopter | socks |
| 飞机 | 苹果 | 牙刷 |  |  | cards | hen | spoon |
| 斧子 | 葡萄 | 烟 |  |  | carrot | horse | swallow |
| 钢笔 | 企鹅 | 眼睛 |  |  | chain | key | swan |
| 胳膊 | 伞 | 洋葱 |  |  | clock | kite | television |
| 公共汽车 | 哨子 | 衣架 |  |  | comb | ladder | tomato |
| 公鸡 | 狮子 | 椅子 |  |  | Compass | leg | train |
| 狗 | 食指 | 樱桃 |  |  | computer | leopard | tree |
| 猴子 | 手 | 鹰 |  |  | corn | Lips | truck |
| 花 | 手套 | 浴盆 |  |  | cow | moon | vest |
| 花瓶 | 书架 | 长颈鹿 |  |  | curtains | mouse | watch |
| 花生 | 书桌 | 猪 |  |  | dragonfly | mushroom | zebra |
| 火箭 | 树叶 |  |  |  | dress | necklace |  |

**Table S3**

*The word frequency of the picture names*

| Name(L1) | Word frequency |  | Name (L2) | Word frequency |
| --- | --- | --- | --- | --- |
| 杯子 | 1.95 |  | arrow | 2.77 |
| 冰箱 | 4.32 |  | balloon | 1.95 |
| 草莓 | 3.56 |  | banana | 2.20 |
| 叉子 | 2.40 |  | basket | 3.22 |
| 插头 | 1.39 |  | bed | 5.14 |
| 茶杯 | 4.13 |  | belt | 3.30 |
| 车轮 | 2.94 |  | bicycle | 1.79 |
| 衬衫 | 3.43 |  | boat | 1.95 |
| 尺子 | 4.52 |  | book | 6.08 |
| 大衣 | 3.30 |  | bread | 4.32 |
| 袋鼠 | 2.64 |  | broom | 2.20 |
| 蛋糕 | 3.66 |  | butterfly | 2.40 |
| 地球 | 1.95 |  | button | 3.30 |
| 电话 | 6.98 |  | candle | 2.83 |
| 房子 | 6.26 |  | car | 5.87 |
| 飞机 | 6.06 |  | cards |  |
| 斧子 | 2.64 |  | carrot | 2.20 |
| 钢笔 | 3.09 |  | chain | 3.89 |
| 胳膊 | 7.32 |  | clock | 3.69 |
| 公共汽车 | 3.00 |  | comb | 1.79 |
| 公鸡 | 5.24 |  | compass |  |
| 狗 | 5.96 |  | computer |  |
| 猴子 | 4.26 |  | corn | 3.22 |
| 花 | 6.99 |  | cow | 3.71 |
| 花瓶 | 3.61 |  | curtains |  |
| 花生 | 3.37 |  | dragonfly |  |
| 火箭 | 3.40 |  | dress | 4.48 |
| 镜子 | 4.75 |  | ear | 4.49 |
| 酒瓶 | 2.64 |  | elephant | 3.22 |
| 拉链 | 0.69 |  | envelope | 3.22 |
| 老虎 | 5.21 |  | fence | 3.43 |
| 铃铛 | 1.39 |  | fish | 5.10 |
| 领带 | 2.77 |  | flag | 3.30 |
| 鹿 | 3.91 |  | foot | 5.79 |
| 蚂蚁 | 4.65 |  | fox | 2.77 |
| 猫 | 5.00 |  | frog | 2.30 |
| 帽子 | 4.69 |  | glasses | 4.98 |
| 门 | 6.63 |  | goat | 3.37 |
| 拇指 | 2.20 |  | guitar | 2.08 |
| 鸟 | 4.26 |  | gun | 4.61 |
| 女孩 | 6.07 |  | hammer | 2.48 |
| 盆 | 5.31 |  | helicopter | 2.83 |
| 苹果 | 4.60 |  | hen |  |
| 葡萄 | 4.16 |  | horse | 4.89 |
| 企鹅 | 2.64 |  | key | 4.47 |
| 伞 | 3.33 |  | kite | 1.79 |
| 哨子 | 2.77 |  | ladder | 2.83 |
| 狮子 | 4.58 |  | leg | 5.17 |
| 食指 | 4.70 |  | leopard | 2.20 |
| 手 | 7.32 |  | lips |  |
| 手套 | 3.40 |  | moon | 4.09 |
| 书架 |  |  | mouse | 2.94 |
| 书桌 | 3.58 |  | mushroom | 2.64 |
| 树叶 | 4.13 |  | necklace | 4.90 |
| 刷子 | 2.30 |  | needle | 2.83 |
| 松鼠 | 3.18 |  | nose | 4.41 |
| 锁 | 3.89 |  | pants | 2.83 |
| 台灯 | 2.08 |  | pear | 1.95 |
| 太阳 | 6.06 |  | pencil | 3.00 |
| 兔子 | 3.66 |  | piano | 3.33 |
| 乌龟 | 3.76 |  | pineapple | 1.39 |
| 五角星 |  |  | pumpkin | 1.10 |
| 五线谱 | 2.20 |  | radio |  |
| 西瓜 | 3.87 |  | scissors | 1.61 |
| 虾 | 2.83 |  | seahorse |  |
| 小丑 | 3.18 |  | seal | 2.71 |
| 熊 | 5.12 |  | shoe | 4.38 |
| 雪人 | 2.64 |  | snail | 1.61 |
| 鸭子 | 3.71 |  | socks | 2.94 |
| 牙刷 | 2.83 |  | spoon | 2.77 |
| 烟 | 4.19 |  | swallow |  |
| 眼睛 | 6.46 |  | swan | 2.08 |
| 洋葱 | 2.56 |  | television |  |
| 衣架 | 1.39 |  | tomato | 2.71 |
| 椅子 | 4.68 |  | train | 4.41 |
| 樱桃 | 2.64 |  | tree | 5.26 |
| 鹰 | 3.14 |  | truck | 3.61 |
| 浴盆 | 3.22 |  | vest | 2.08 |
| 长颈鹿 | 2.83 |  | watch | 3.71 |
| 猪 | 5.26 |  | zebra | 1.10 |

**Table S4**

A summary of the language control patterns across the three contexts

| Session | Reactive language control | Proactive language control |
| --- | --- | --- |
| Baseline session | Similar local inhibition:  N2：No significant difference between L1 switching and L2 switching  LPC: No significant difference between L1 switching and L2 switching | Greater global inhibition for **L1:**  Reversed language dominance;  LPC: L2>L1 |
| 25% face-language matched Session | Greater local inhibition for **L2:**  Switch cost: L2>L1;  N2: L1 switching>L2 switching  LPC: L1 switching>L2 switching | Greater global inhibition for **L1:**  Reversed language dominance;  LPC: L2>L1 |
| 75% face-language matched Session | Greater local inhibition for **L1**:  Switch cost: L1>L2;  N2: L2 switching>L1 switching  LPC: L2 switching<L1 switching | Greater global inhibition for **L1**:  Reversed language dominance;  LPC: L2>L1 |
